# The Physiology Of the WEight Reduced State (POWERS) study: assessing energy balance

**DOI:** 10.1038/s41366-025-01935-x

**Published:** 2025-11-17

**Authors:** Michael Rosenbaum, Kelly C. Allison, Maren R. Laughlin, Kathryn Whyte, John M. Jakicic, Laurel E. S. Mayer, Maxine Ashby-Thompson, Matthew R. Hayes, John Speakman, Rudolph L. Leibel, Sai Krupa Das, Dympna Gallagher, Susan B. Roberts, POWERS Consortium

**Affiliations:** 1 Department of Pediatrics, Division of Molecular Genetics, Columbia University Irving Medical Center, New York, NY, USA.; 2 Department of Medicine, Division of General Medicine, Columbia University Irving Medical Center, New York, NY, USA.; 3 Department of Psychiatry, Perelman School of Medicine, University of Pennsylvania, Philadelphia, PA, USA.; 4 Division of Diabetes, Endocrinology and Metabolic Diseases, National Institute of Diabetes and Digestive and Kidney Diseases, National Institutes of Health, Bethesda, MD, USA.; 5 Department of Internal Medicine, Division of Physical Activity and Weight Management, University of Kansas Medical Center, Kansas City, KS, USA.; 6 Department of Psychiatry, Columbia University Irving Medical Center, New York, NY, USA.; 7 Division of Endocrinology, New York Nutrition Obesity Research Center, Department of Medicine, Columbia University, New York, NY, USA.; 8 School of Biological Sciences, University of Aberdeen, Aberdeen, UK.; 9 Energy Metabolism Team, Jean Mayer USDA Human Nutrition Research Center on Aging, Gerald J. and Dorothy R. Friedman School of Nutrition Science and Policy, Tufts University, Medford, MA, USA.; 10 Dartmouth College Geisel School of Medicine, Hanover, NH, USA.; 26 These authors contributed equally: Michael Rosenbaum, Kelly C. Allison.; 27 These authors jointly supervised this work: Sai Krupa Das, Dympna Gallagher, Susan B. Roberts.

## Abstract

**BACKGROUND/OBJECTIVES:**

We provide the rationale for and description of energy balance measures (i.e., energy intake and energy expenditure) in The Physiology Of the WEight Reduced State (POWERS) study which aims to understand the contribution of the many factors that influence weight regain following behavioral weight loss.

**METHODS:**

The primary dependent variable is weight regain over 1 year following a 7% or greater supervised weight loss. The balance between energy intake and expenditure is the primary determinant of weight regain. Healthy adults (target *n* = 205), aged 25- < 60 years, with body mass index (BMI) 30- < 40 kg/m^2^ are being recruited. Energy intake and expenditure phenotypes are measured prior to weight loss (baseline, BL), immediately following weight loss (T0), and then four (T4) and 12 months (T12) after weight loss. Weight stability is required before BL and T0 measurement periods. Weight change at T12 from T0 is the primary outcome variable. Energy intake is measured with serial doubly labeled water (DLW) measurements combined with dual x-ray absorptiometry (DXA) to assess changes in fat and lean mass; DLW is also used to measure twenty-four-hour energy expenditure (TEE). Components of TEE including resting energy expenditure (REE) and non-resting and activity energy expenditure (NREE and AEE), as well as skeletal muscle chemomechanical efficiency and grip strength are assessed. Self-reported dietary intake is assessed with interviewer-administered multiple-pass 24-hour food recalls.

**DISCUSSION:**

This manuscript describes the rationale for the methods chosen to assess energy balance and the analytical methods employed to normalize and express data in the setting of changes in body weight and composition immediately following behavioral weight loss and thereafter at 4- and 12-months post-weight loss.

## INTRODUCTION

The primary goal of the Physiology Of the WEight Reduced State (POWERS) study is to determine the physiological basis for the variability in weight regain among adults with obesity following clinically significant weight loss achieved in a behavioral weight loss (BWL) intervention including diet, exercise, and regular selfmonitoring. The rationale for, and overall design of POWERS are provided elsewhere [1]. The physiological contributors to energy balance are examined to understand factors that differentiate those who maintain their reduced weight, continue to lose weight, or regain some or all their lost weight. Adaptive thermogenesis [declines in energy expenditure (EE) beyond those attributable to changes in body composition or time spent in physical activity (PA)] and increased energy intake (EI) reported to occur with BWL have been hypothesized to play a role, but have not been significantly correlated with subsequent weight [2-4]. No studies have examined the effects of early changes occurring in EI and EE during weight maintenance efforts following BWL.

Changes in weight, and in energy stores [sum of energy in changes in fat mass (FM) and fat-free mass (FFM)] over time must reflect differences between daily EI and EE (TEE) [5, 6], POWERS’ primary independent variables. These factors will be measured at four points: Baseline (BL, prior to a BWL intervention); T0 (directly following weight loss of ≥7% and 2 weeks of weight stability); T4 (4 months following T0), and T12 (12 months following T0). No intervention will be provided after T0. Data will be analyzed to examine the effects on daily EI and EE, both calculated as an average of 14 days (see below) and weight regain of weight loss of ≥7% of BL (changes from BL to T0), and the early phase (T0 to T4) and the later phase (T4 to T12) of weight change occurring without behavioral support from T0 to T12. The primary and secondary measures of dependent and independent variables related to energy balance are summarized in Table 1.

Average responses to BWL interventions are weight loss (approximately 4–9 months), then relative weight stability (approximately 5–9 months), then weight regain. POWERS will study the variability in these responses to understand the predictors and drivers of changes in body weight and energy stores after weight loss [7-12]. Weight stability will be required before BL and T0 study points to ensure these crucial measures are not influenced by acute effects of negative or positive energy balance. T4 was chosen to maximize the opportunity to examine detectable changes in EI and TEE in the early period following weight loss (when significant weight change from T0 is less likely) that may be premonitory of subsequent weight change. T12 measurements allow observation of EI and EE over time and study of temporal correlations of early and later measures after weight loss.

The relationship of body composition, EI and EE is complex. FFM is the primary determinant of TEE, especially resting energy expenditure (REE). Fat mass (FM) has a larger effect on activity energy expenditure (AEE) than REE due to the extra work involved in moving a total body mass when engaged in PA [13, 14]. Changes in energy balance (weight gain or loss) and energy stores (predominantly FM) affect these relationships.

**Fig. 1:**
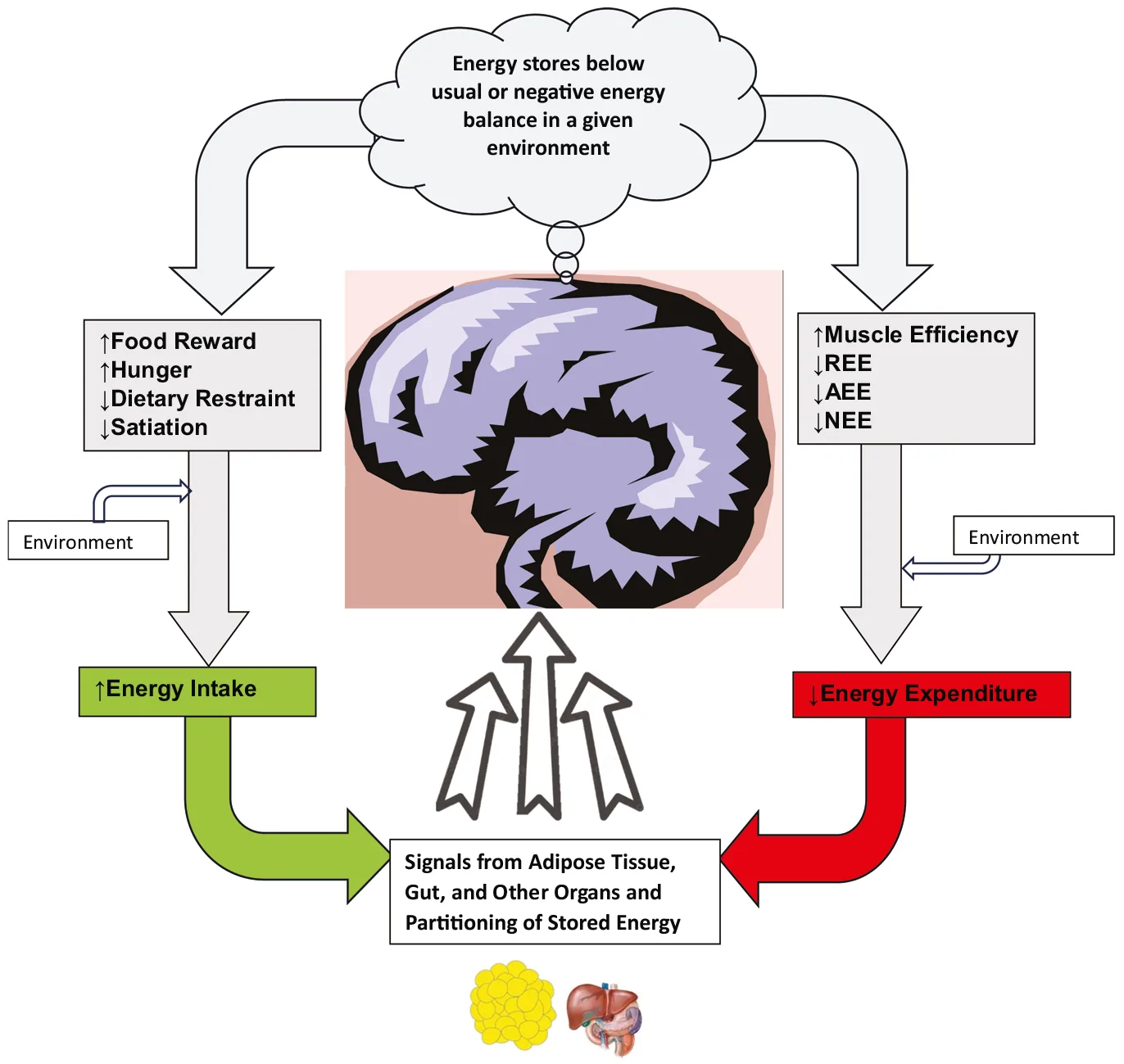
Schematic of the systems regulating body weight. These systems act coordinately to increase energy intake (EI) and decrease energy expenditure (EE) in response to negative energy balance or reduced energy stores (predominantly fat). The degrees to which various afferent signals of energy stores (predominantly from adipose tissue) and efferent outputs affecting both EI and EE (predominantly originating in the central nervous system) are “engaged” during and following weight reduction are highly variable between individuals which contributes to the large inter-individual variability in weight regain over longer timeframes. In POWERS, interrogating this variability will advance understanding of the physiology of the weight-reduced state.

At usual weight, EI and TEE vary directly to contribute to maintenance of body weight despite wide daily fluctuations in diet and PA. This apparent “coupling” is consistent with the relative constancy of body weight in U.S. adults with average gain of 0.5 to 1.5 kg/year (about 4000 kcal, less than 0.5% of the average annual caloric intake) [15-17]. Attempts to lose weight (negative energy balance) and avoid regain (maintain energy balance) have been associated with varying degrees of increased appetite and decline in TEE [18-20] beyond those predicted by changes in body weight and energy stores (see Fig. 1). Data suggest EI may change in response to variation in TEE [21] more than EE responds to changes in EI. REE and AEE adjusted for age and body composition have been reported to account, respectively, for about 55% and 20% of the variation [22-26] in daily EI [27]; short-term changes in EI have much smaller effects on EE [28-32].

POWERS analyzes the nature and regulation of the relationship between EI and EE following weight loss (which are associated with different genotypes [33-35] and phenotypes [36-44], and treatment responses [45-49] than during weight loss). A key purpose of POWERS is to examine this variability under standardized conditions and determine to what extent changes in components of energy balance [50-53] occurring during weight loss and novel changes following weight loss, are associated with subsequent weight regain. This manuscript discusses the methods used to assess energy intake, utilization, stores, and expenditure.

## PRIMARY DEPENDENT AND INDEPENDENT VARIABLES RELATED TO ENERGY BALANCE

The primary outcome (dependent) variables of POWERS are percentage and absolute changes in body weight (kg) over 1 year following a > 7% weight loss. The fractional contributions of changes in FM and FFM to changes in body weight are influenced by many factors including, but not limited to, sex, age, energy balance, diet composition, and PA [54]. FM and FFM must be measured both during and after the weight loss phases to assess fuel partitioning and energy stores (FM and FFM) due to the roughly 9-fold differences in the caloric storage density of FM (9.3 kcal/gm) versus FFM (1.1 kcal/gm) [55]. Comparisons of EI and TEE, REE, AEE and various biomarkers, between participant groups (e.g., ethnicity/race) or within participants (e.g., temporal changes) must be “normalized” to body composition, which will be measured by dual energy x-ray absorptiometry (DXA) for accurate interpretation [56-59].

## ENERGY EXPENDITURE

### Total energy expenditure (TEE)

TEE includes REE, the thermic effect of feeding (TEF), NREE [total daily energy expenditure above resting which consists of (TEF) and AEE (energy expended in PA)] [13], with a variable degree of change observed in EE during and following weight loss [60-62].

The primary measure of TEE in POWERS will be by doubly labeled water (DLW) [63] which relies on the fact that the oxygen in metabolically produced CO_2_ quickly reaches isotopic exchange equilibrium with that in body water due to the action of carbonic anhydrase. CO_2_ production will be measured from the differential elimination rates of deuterium (eliminated only in water) and oxygen (^18^O—eliminated in both water and CO_2_). In the fasted state, a urine sample will be obtained to assess background isotope enrichment. Participants are weighed and given an accurately weighed dose of enriched water to drink with approximately 10 atom percent excess (APE) ^18^O (H_2_^18^O) and 5 APE deuterium (D_2_O) and is calculated to result in an enrichment of about 200 ppm ^18^O excess above background levels and 100 ppm deuterium excess. The dose equilibrates in the body water for the next 3 h during which participants refrain from food or excessive water consumption; additional urine samples are obtained on-site. Participants then provide additional daily urine samples for another 14 days.

The isotope enrichments in urine are determined using off-axis laser spectroscopy [64] in parallel with simultaneously run laboratory and international standards and background enrichment values are subtracted from post-dose samples. The resultant excess enrichments are log_e_ converted, and a linear relationship fitted to the derived values to estimate the elimination constants (*k*_o_ and *k*_d_) (gradients) and back extrapolated to intercepts at the dosing time to estimate the dilution spaces of the two isotopes (N_o_ and N_d_). The values of elimination constants and dilution spaces are then combined to estimate the CO_2_ production using a recently derived equation that performed best in retrospective validation studies [65]. The average accuracy across *n* = 61 simultaneous indirect chamber calorimetry trials for this equation was −0.4% and precision was 7.67%.

To calculate EE, the CO_2_ production must be converted using an estimate of the respiratory exchange ratio [RER the ratio of the volume of CO_2_ produced (VCO_2_) to the volume of O_2_ consumed (VO_2_)]. If the individual is in energy balance (weight stable) this can be derived from the “food quotient,” (FQ) from food intake records. During weight loss or gain this requires adjustment for the part of the expenditure derived from body fat. Similarly, if the individual is in energy balance the expenditure will be equivalent to the metabolizable energy intake but if not, the metabolizable energy intake estimate needs adjustment for the estimated weight change. The main advantage of DLW is that, unlike chamber calorimetry, it reflects TEE in a free-living participant over longer periods of time (usually 7-14 days). The disadvantages relate to imprecision in the conversion of CO_2_ production into EE which depend on the weight status of the participant and the accuracy of the FQ estimate.

### Resting energy expenditure (REE)

Measurement of REE is required to determine NREE and AEE. REE will be measured at all four time points by indirect calorimetry using the same within site-specific equipment throughout the study to minimize error within and between participants. REE measurement is different between the two POWERS sites. Chamber calorimetry has the highest precision and accuracy for REE based on available methodologies [66], but was a resource available only at the Columbia University Irving Medical Center (CUIMC) site. It was decided to use the best REE methodology available at each site, recognizing that the different methodologies will be a limiting factor in interpreting REE data. We aim to collect all within-site and within-participant REE data on the same cart (Philadelphia) and in the same chamber (CUIMC) to maximize accuracy for data being collected within each site. During data analysis, using the sites’ merged data, site will be included as a covariate to ascertain whether differences related to REE instrument are apparent. Methodology by site is described below.

REE will be measured in the morning (approximately 6:30–8:00 a.m.) after an overnight inpatient stay to standardize fasting and other physiological conditions including PA. Prior to REE testing, participants are asked to maintain their usual dietary and activity behaviors during the 72 h prior to clinic visit, with no exercise during the 24-h before the overnight admission [67]. EI, sleep, PA, and menstrual phase (when relevant) are recorded. After a 10–12 overnight fast, the participant rests, but remains awake, on a recliner or in their hospital bed, in a thermoneutral noise-free environment for a measurement period of approximately 45–60 min.

### REE at Columbia University Irving Medical Center (CUIMC)

Data collection will occur using a whole-room calorimeter consisting of an air-tight temperature-controlled room with prespecified flow rate connected to a pull through calorimetry system (Sable Systems, Las Vegas NV). By flowing a known amount of fresh air through the room, respiratory gases from the study participant are sampled on the exhaust side of the system for measurement of O_2_ and CO_2_ using fuel cell oxygen and near infrared carbon dioxide sensors (Model GA-3m2, Sable Systems Intl, Las Vegas, NV) and analysis software for a SW-Promethium System is installed on a Sable Systems approved computer, with a Sable Systems Promethium Interface Module (Model IM-2). Data are recorded and processed on-line by the Sable software programs Caloscreen and Expedata. During the 60-min data collection period, the participant will be asked to remain motionless, awake, and relaxed. The first and last 10 min of data collection are discarded to allow for the room and sampling line air to reach steady state and eliminate anticipatory signaling from the participant that may artificially inflate EE. A more robust REE will be calculated from the expiratory gases collected in steady state during the remaining 40 min.

### REE at Drexel-Dartmouth-Tufts-Penn (DTP)

REE will be measured via indirect calorimetry using a ventilated hood system (Parvo TrueOne 2400, http://www.parvo.com/trueone-2400/). Participants are asked to remain supine in bed, motionless, awake, and relaxed for the 45-min test. The first 15 min of test data are discarded. Calculations of O_2_ consumption ($\dot{V}O_{2}$) and CO_2_ production ($\dot{V}{\text{CO}}_{2}$) for the subsequent 30 min are generated from continuous measurements of gas exchange and used to calculate rate of REE. The calorimeter will be calibrated before each measurement with standardized gases and room air, and measurements of $\dot{V}O$, $\dot{V}\text{CO}$ and respiratory quotient (RQ) are required to be within 2% of calibration values.

REE at both sites will be calculated using the Weir formula [68] to convert the steady state concentrations of collected respiratory gases ($\dot{V}O_{2}$ L/min and $\dot{V}{\text{CO}}_{2}$ L/min)) to EE in kilocalories per min (kcal/min) [REE, kcal/min = 3.941($\dot{V}O_{2}$) + 1.106($\dot{V}{\text{CO}}_{2}$). The coefficient of variation for REE is 2.8% ± 2.0% kcal/h, 3.1% ± 2.0% L/min for $\dot{V}O_{2}$ and 4.1% ± 1.7% L/min for $\dot{V}{\text{CO}}_{2}$.

*NREE and AEE* reflect, respectively, all energy expended above resting and all energy expended in PA. They are calculated as:

NREE=TEE−−REE

AEE=NREE−−TEF

TEF (thermic effect of feeding), calculated as a percentage (10%) of EI derived from DLW, constitutes a small fraction of TEE and, and has not been shown to be affected by weight loss [69]. The inter-individual TEF variability is anticipated to result in a much greater variation in AEE compared to NREE even corrected for body composition and time spent in PA [70] which is measured by actigraphy during the DLW period (see below).

## ADAPTIVE THERMOGENESIS

Adaptive thermogenesis is defined as the decline in TEE and its components occurring during and/or following weight loss [19] that is independent of body weight and composition [71] and not accounted for by changes in total PA. At any single point EE is often expressed per unit of FFM (TEE and REE), or weight (NREE and AEE). These ratios are not appropriate for longitudinal studies such as POWERS because the regression lines of EE measures to FFM or weight have non-zero intercepts, partially due to varying energy requirements of different FFM components. This issue is addressed by calculating the difference (residual) between predicted and actual EE for participants at each time-period, calculated as the measured EE values minus the predicted value for each participant based on regression of all EE values (TEE, REE, or AEE) against all relevant weight and body composition values (i.e., FFM and FM) in the same cohort measured at BL [4]. This will allow for temporal measurement of adaptive thermogenesis due to BWL, and duration of that adaptation up to a year (see Table 2).

Controlled in-patient studies with prescribed PA show that skeletal muscle chemomechanical contractile efficiency (MCME) is significantly associated with adaptive thermogenesis in NREE [72, 73]. There is, on the average, a significant increase in the amount of time spent in PA during and following BWL which is even greater if the weight loss intervention includes exercise as in the present study [74]. Changes in time spent in PA during the study are potential confounders of calculations of adaptative thermogenesis in TEE, NREE, and AEE. To minimize confounding effects of time spent in PA (behavioral), but not spontaneous or isometric PA, on assessments of physiological changes in thermogenesis, calculating changes in TEE, NREE and AEE will include the time spent in PA, which is expected to be light to moderate intensity, measured by accelerometry as a covariate. In addition to time spent in PA, there may also be BWL effects on energy expenditure arising from spontaneous physical activity, such as fidgeting and maintaining posture, and resting muscle physical activity to maintain body temperature, which may also contribute to adaptive thermogenesis but are not assessed in this study [75].

## ENERGY INTAKE (EI)

EI is a core variable and is defined as consumed metabolizable energy, assuming standard coefficients for the ratio of metabolizable energy to gross energy content [76]. Dietary quality with respect to macronutrients, micronutrients, dietary fiber, food form, energy density, portion size, and glycemic load will also be analyzed for additional contributions to energy balance. Assessments of components of EI, such as hunger, satiety, nausea, food cravings, disinhibition, and restraint are measured during various eating tasks, described elsewhere [77].

The primary measure of EI in POWERS will be based on changes in body energy stores over the 14 days when TEE is measured by DLW [55]. DXA measurements are performed on Day 1, immediately following ingestion of DLW isotopes, and again on Day 15, the final urine collection day. Changes in energy stores (ES) from DXA are calculated as follows:

ΔES(kcal∕day)=[ΔFM(g∕day)×9.3kcal∕g]+[ΔFFM(g∕day)×1.1kcal∕g]

EI over the 14-day period will be calculated as the sum of TEE and the change in energy stores. We plan to average EI during T0–T12 by integrating TEE over that period from DLW data and summing TEE with changes in body energy stores, which will reduce errors associated with short-term measurements.

## SECONDARY INDEPENDENT VARIABLES IN POWERS

### Body composition

DXA measures FM, FFM and bone mineral content (BMC), where FFM represents the sum of lean mass (LM) and BMC at whole-body and regional (trunk, arms, legs, android, gynoid) levels [78-80]. At each study site, measures are collected on the same whole-body scanner (iDXA, GE Healthcare, Madison, WI; Encore software v16 at CUIMC and Horizon model, Hologic Inc., Marlborough, MA; software 13.5.3.1:3 at DTP). Eight DXA scans within 18 months are well within acceptable radiation safety limits. A DXA study identifies aggregate FFM which will be treated as a single variable in subsequent analyses but does not allow assessment of the contributions of individual components of FFM (muscle, liver, brain, etc.) which have significantly different metabolic rates [81, 82]. It should also be noted that the relative contributions of individual components of FFM to energy expenditure, as well as the hydration status of these components, may be affected by changes in body weight [83] and thereby influence interpretation of DXA data as it affects calculation of EE (reflecting changes in assessment of body composition) and EI (reflecting changes in both body composition and the energy density of FM and FFM).

Participant's maximum body width will be measured to confirm that all body parts fit within the DXA field-of-view, and all individuals of child-bearing potential must have a negative urine pregnancy test before each scan. Participants wear a hospital gown and are positioned with legs fully extended and flat, with hips and shoulders level, the spine straight, and the chin line above the shoulders. Arms are extended with hands turned in with thumbs facing up. Heels are separated by 6-10 inches and the legs inwardly rotated with toes touching. A short X-ray transparent Velcro strap (or masking tape) may encircle the top of the feet for proper positioning.

Reproducibility of DXA in adults is ±3.4% for FM and ±1.2% for FFM [84-86]. Scan modes are age and body weight/size-appropriate based on the manufacturer’s recommendations. All scans will be analyzed using the same software version at study completion. For quality control, an anthropomorphic spine phantom made of calcium hydroxyapatite embedded in a 17.5 × 15 × 17.5 cm block will be scanned each morning prior to study, and before and after all DXA system maintenance visits. A protocol for calibration between sites will be established.

### Waist circumference

Waist circumference is a secondary independent variable in POWERS because of its relationship to other secondary outcome variables including hypertension, fatty liver disease, and insulin resistance. It will be also used as a “proxy” for abdominal fat and measured at all time points (BL, T0, T4 and T12) using a springloaded Gulick II Measuring Tape (to allow consistent tension) at a marked point mid-way between the iliac crest and lowermost rib on bare skin. Participants will be instructed to breathe normally while staff take the measurement twice, with confirmation that the tape remained horizontal. If the measurements are within 0.5 cm, they are averaged. If not, a third will be taken. If all three are discrepant by >0.5 cm, the participant will be remeasured by another trained staff member.

### Skeletal muscle chemomechanical efficiency (MCME)

Activity energy expenditure (AEE) is the compartment of EE proposed to account for most of the variability in changes in EE after weight loss [87], most likely due to an approximately 20% increase in MCME at low levels of exercise (commensurate with activities of daily living) which is significantly correlated with the residual in NREE [72, 73, 88, 89] independent of changes in time spent in PA [90]. MCME is defined as the amount of power (watts) above resting generated per volume of oxygen consumed during pedaling on a bicycle ergometer at incremental levels of resistance [73, 87].

Comparison of MCME at BL to T0 provides information regarding the relationship of MCME to energy expended in PA, which may affect subsequent weight regain and changes in MCME after weight loss. Comparisons of MCME from T0 to T4 allow investigation of whether changes in MCME during the early months of unsupervised weight maintenance correlate with weight regain from T0 to T4 and from T0 to T12. Comparisons of MCME from T0 and T4 to T12, respectively, allow investigation of whether the changes in MCME from BL to T0 or T0 to T4 are predictive of changes in later weight regain. Using all time points, the relationship of changes in MCME to energy expended in PA will be investigated, as weight changes.

MCME will be evaluated by standardized incremental graded cycle ergometry an electronically braked cycle ergometer (Lode Corival). Using indirect calorimetry (Parvo Medics TrueOne 2400 metabolic cart), O_2_ consumption and CO_2_ production values are collected during rest while seated on the cycle ergometer (4 min); thereafter, during exercise performed at specific incremental intensities: 10 Watts (4 min), 25 W (4 min) and 50 Watts (4 min). Based on existing data [72, 73, 87, 91], most effects of weight loss on MCME should be evident at 10 and 25 W. The MCME at 50 W serves to ascertain whether the measurable effects of weight loss on MCME have been detected at the lower levels of work. A final stage at 75 W (2 min) will be performed to allow estimation of cardio-respiratory fitness. Heart rate will be measured throughout the test.

Skeletal muscle contractile efficiency reflects skeletal muscle gene expression and biochemistry [73, 87, 91] as well as mitochondrial function. These will be assessed in skeletal muscle samples taken by needle biopsy at T0 and T4 [92], and will provide insights into how weight regain affects the relationship between MCME and skeletal molecular physiology.

### Isometric hand grip strength

Hand grip strength is a commonly used measure of upper body skeletal muscle function [93, 94] and has been reported to decrease following weight reduction [95]. It is not known to what degree changes in grip strength reflect changes in muscle composition MCME [91]. Grip strength in both hands will be measured as according to the Lifestyle for Independence in Elders trial (LIFE) [96] using a JAMAR handheld hydraulic dynamometer (Patterson Medical) at all study periods (BL, T0, T4, T12). This test will be performed in duplicate in a seated position, arm resting on a table with the elbow bent. The participant will be instructed to squeeze as hard as they can, allowing 10 seconds between each measurement. This tests the potential change in grip strength with weight loss and across the maintenance phase and associations between change in grip strength and change in appendicular lean mass (measured by DXA).

### Total daily energy intake and diet composition

The primary assessment of EI described above estimates caloric intake but not diet composition. The multiple pass *24-h recall method* [97] assesses average total daily food intake (both quantity and quality) in a more naturalistic setting and permits assessment of the possible effects of the differences in perceived vs. actual intake on subsequent weight regain [98].

Four 24-h dietary recalls over a 2-week period are conducted at BL, T0, T4, and T12. One is conducted the day before REE measures, to provide information on dietary intake in the immediate period before REE. The other three recall timepoints are selected at random, with the goal of obtaining two on weekdays and one on weekends, capturing potential variability in dietary intake and quality [99]. Additional 3-day dietary assessments are collected monthly during the T0 to T12 period.

The recalls are analyzed using the latest version of Nutrition Data System for Research (NDSR, Nutrition Coordinating Center (NCC), University of Minnesota, Minneapolis, MN). Output data include daily reported EI, macronutrient (fat, protein, carbohydrate) intakes, daily time interval for eating, number of meals and snacks, food groups, diet quality, diet variety, and dietary supplement use. The 24-h recall data are also used to calculate a Healthy Eating Index score using algorithms and SAS code provided by the University of Minnesota NCC and the National Cancer Institute. Direct observation of energy intake during a single meal and energy intake in the absence of hunger are being collected at T0 and T4 and are described elsewhere [77].

## CONCLUSIONS

The methods of assessing energy intake, stores, and expenditure being employed in POWERS reflect the application of state-of-the-art methodology to studies of energy balance in communitydwelling participants. The physiological, genetic, environmental, behavioral, cognitive, and other –omics that affect these variables are discussed in other papers in this issue. Both the changes in EI and EE that occur with weight loss (baseline to T0) and during the early post-weight loss phase (T0 to T4) are examined in POWERS and related to the multiple mechanistic and behavioral variables discussed in other papers in this issue, to create a systems model of body weight regulation that can be leveraged to optimize interventions to promote weight loss maintenance and prevent weight regain.

## Figures and Tables

**Table 1.** Summary of measures.

Variable	Primary Measure	Secondary Measures
**Weight**	Body weight (scale in clinic)	Fat mass (dual energy X-ray absorptiometry, DXA), fat-free mass (DXA), height, waist circumference, body mass index (BMI)
**Energy Expenditure**	Total energy expenditure [doubly labeled water (DLW)]	Resting energy expenditure (REE), non-resting and activity energy expenditure (NREE and AEE), bicycle ergometry, grip strength
**Energy Intake**	Calculated from DLW and DXA	24-hour food recalls

Non-resting energy expenditure (NREE) will be calculated as the difference between TEE and REE. Activity energy expenditure (AEE) will be calculated as the differences between NREE and the thermic effect of feeding (TEF, 0.1 x TEE).

Resting energy expenditure (REE) will be measured by indirect calorimetry.

**Table 2.** Calculations of residuals.

Residual	Calculation
**Weight Loss**	*(Predicted value at T0 from baseline data) – (Measured T0 value)*
**Early Weight Regain**	*(Predicted value at T4 from T0 data) – (Measured T4 value)*
**Late Weight Regain**	*(Predicted value at T12 from T4 data) – (Measured T12 value)*
**Overall Weight Regain**	*(Predicted value at T12 from T0 data) – (Measured T0 value)*
**Overall Study**	*(Predicted value at T12 from baseline data) – (Measured T12 value)*

## FOR THE POWERS CONSORTIUM

**CUIMC (CLINICAL CENTER)** Dympna Gallagher^11^, Rudolph Leibel^11^, Laurel Mayer^11^, Michael Rosenbaum^11^, Maxine Ashby-Thompson^11^, Giada Benasi^11^, Karin Foerde^11^, Rochelle Goldsmith^11^, Michio Hirano^11^, Charles LeDuc^11^, Christina Roberto^11^, Heather Seid^11^, Marie-Pierre St-Onge^11^, Kathryn Whyte^11^, Yiying Zhang^11^, Alexis O. Aparicio^11^, Daaimah Dratsky^11^, Jingrui Gu^11^, Michelle Horowitz^11^, Susan Xiaoqin Lin^11^, Arden McMath^11^, Cynthia Mikula^11^, Joel Matos Nunez^11^, Martin Picard^11^, Janet Schebendach^11^, Yifei Sun^11^, Agnes Wong^11^, Wen Wen Yu^11^ and Bret Goodpaster^12^

**DTP (CLINICAL CENTER)** Susan B. Roberts^13^, Michael Lowe^14^, Alexis Gomez^14^, Sophie Meierovich^14^, Rachel Saks^14^, Mars Scharf^14^, Edward Williams^14^, Anna Zhou^14^, Olive Zhu^14^, Kelly C. Allison^15^, Matthew R. Hayes^15^, Michael Rickels^15^, David Roalf^15^, Gary Wu^15^, Payman Zamani^15^, Lillian Chau^15^, Adam Czernuszenko^15^, Cassandra Demastus^15^, Melissa Fernando^15^, Kubarah Ghias^15^, Gabrielle Grosso^15^, Lindsay Herman^15^, Nathaniel Holmes^15^, Christina Mastracchio^15^, Varsha Sayana^15^, Nicholas Wellman^15^, Sai Krupa Das^16^, Roger Fielding^16^, Andrew Howland^16^, Kyle Burger^17^, John Speakman^18^ and Catherine Hambly^18^

**DATA COORDINATING CENTER** Steven H. Belle^19^, Wendy C. King^19^, David Hallam^19^, Tamara Haller^19^, Stephanie S. Kelley^19^, Christopher E. Kline^19^, Kelsey R. Leonard^19^, Andrew J. Pelesko^19^, Matthew Zourelias^19^, Panayiotis V. Benos^20^, John M. Jakicic^21^, Abdus S. Wahed^22^ and Kirk I. Erickson^12^

**NIH** Maren R. Laughlin^23^, Susan Yanovski^23^, Bramaramba Kowtha^24^ and Deborah Young-Hyman^25^

^11^Columbia University, New York, NY, USA. ^12^AdventHealth Research Institute, Orlando, FL, USA. ^13^Dartmouth College, Hanover, NH, USA. ^14^Drexel University, Philadelphia, PA, USA. ^15^University of Pennsylvania, Philadelphia, PA, USA. ^16^Tufts University, Boston, MA, USA. ^17^University of North Carolina at Chapel Hill, Chapel Hill, NC, USA. ^18^University of Aberdeen, Glasgow, Scotland, UK. ^19^University of Pittsburgh, Pittsburgh, PA, USA. ^20^University of Florida, Gainesville, FL, USA. ^21^University of Kansas Medical Center, Kansas City, KS, USA. ^22^University of Rochester, Rochester, NY, USA. ^23^National Institute of Diabetes and Digestive and Kidney Diseases, Bethesda, MD, USA. ^24^Office of Disease Prevention, Bethesda, MD, USA. ^25^Office of Behavior and Social Sciences Research, Bethesda, MD, USA.
